# The Role of Multicancer Early Detection Tests in Head and Neck Squamous Cell Carcinoma: A Case Series

**DOI:** 10.1002/hed.70337

**Published:** 2026-05-28

**Authors:** Abhinav Talwar, Olivia Ben‐Levy, Nikhil Sriram, Sandeep Samant

**Affiliations:** ^1^ Department of Otolaryngology‐Head and Neck Surgery Northwestern Memorial Hospital Chicago Illinois USA; ^2^ Feinberg School of Medicine Chicago Illinois USA

**Keywords:** Galleri, head and neck cancer, MCED

## Abstract

**Background:**

Head and neck squamous cell carcinoma (HNSCC) lacks an established screening program, and most cases are diagnosed at advanced stages. Multi‐cancer early detection (MCED) blood‐based assays represent a promising approach, yet real‐world clinical experience with MCED‐identified HNSCC remains limited.

**Methods:**

We retrospectively reviewed patients referred to our Otolaryngology department with a positive Galleri MCED result indicating HNSCC between January 2020 and March 2026. Physical exam findings, flexible laryngoscopy findings, imaging, and treatment data for each patient was reviewed.

**Results:**

Three patients were included in this case report. All three patients were diagnosed with early‐stage p16+ oropharyngeal squamous cell carcinoma despite absent symptoms and largely unremarkable physical examinations. All underwent successful resection with no evidence of recurrent disease at early follow‐up.

**Conclusions:**

MCED testing can potentially identify early‐stage HNSCC in asymptomatic patients. Otolaryngologists should be prepared to systematically evaluate patients referred following a positive MCED result.

## 1 Introduction

Head and neck squamous cell carcinoma (HNSCC) represents a growing global health burden without an established population‐level screening program. Survival is highly stage‐dependent, with 5‐year rates ranging from 70%–90% for early‐stage disease to 25%–60% for locoregionally advanced disease and less than 20% for metastatic disease [1]. This stark survival gradient underscores the critical importance of early detection.

Development of screening tools for HNSCC is challenging for several reasons. The low prevalence of HNSCC in the general population, heterogeneous disease states across multiple anatomic sites, and the absence of a single accessible target organ or precursor lesion all complicate screening program design. The anatomic inaccessibility of common HNSCC primary sites to routine physical examination also poses a significant barrier to early detection. Tumors arising in the nasopharynx, base of tongue, supraglottic larynx, and hypopharynx often remain undetected without specialized fiberoptic examination, resulting in late‐stage presentation and poor clinical outcomes [2].

This diagnostic gap has driven interest in novel, minimally invasive approaches to cancer detection. Liquid biopsy strategies, which analyze tumor derived material circulating in the bloodstream, have emerged as a promising avenue for early detection for malignancy. In HPV‐positive oropharyngeal cancer specifically, commercially available circulating tumor DNA (ctDNA) tests such as NavDx (Naveris, Waltham, MA, USA) have demonstrated clinical utility for surveillance purposes and are especially beneficial for screening in high‐risk populations [3]. However, new tests on the horizon purport to move beyond single‐cancer identification, screening for many cancer types in a single assay. These so‐called multi‐cancer early detection (MCED) tests are blood‐based assays that analyze circulating cell‐free DNA (cfDNA). One such MCED test, Galleri (GRAIL, Menlo Park, CA, USA), has garnered attention for its potential to screen across a wide range of cancer types by leveraging circulating DNA methylation patterns [4]. Early institutional experience at Dana‐Farber Cancer Institute demonstrated that among 14 patients presenting with a positive Galleri result, 78.6% had a confirmed cancer diagnosis [5]. Further real‐world validation of the test across more than 100 000 individuals confirmed a cancer signal detection rate of 0.91% with 74% of detected cancers lacking established U.S. Preventive Services Task Force (USPSTF)‐recommended screening [6].

Kennedy et al. published a state‐of‐the‐art review of MCED testing for otolaryngologists, highlighting that Galleri demonstrates among the highest sensitivity for head and neck cancer, and emphasizing that otolaryngologists are increasingly serving as the recipients of referrals following positive MCED results [7]. To date, the published clinical experience with MCED‐identified HNSCC remains extremely limited. Real‐world, nonindustry‐sponsored clinical experience with MCED‐identified HNSCC consists of just five reported patients. Of these five patients, two were confirmed to have HPV‐positive oropharyngeal squamous cell carcinoma [8]. This paucity of real‐world data represents a critical gap at a moment when MCED testing is rapidly entering clinical practice. In this study, we describe three additional patients with HNSCC identified through the Galleri test at an independent institution, contributing to the nascent but growing body of evidence characterizing the clinical utility and diagnostic implications of MCED technology in HNSCC.

## 2 Case Reports

This study was determined to be exempt from full review by the institutional review board. We conducted a retrospective review of patients referred to our institution's Otolaryngology Head and Neck Surgery department with a positive Galleri MCED result suggesting a diagnosis of HNSCC from January 2020 to March 2026. We collected data on clinical presentation, MCED test results, post‐MCED diagnostic workup, diagnosis, treatment, and clinical outcomes.

Three patients met inclusion criteria for this review. They were all referred to otolaryngology for a positive MCED indicating HNSCC. The mean age of the patients on presentation was 59 years. Patient clinicodemographic as well as treatment/outcome data are presented in Table 1.

**TABLE 1:** Clinicodemographic, treatment, and pathologic data of three included patients.

Pt	Age	Sex	Pre‐MCED concerns	MCED result	Post‐MCED physical exam	Imaging/Workup	Treatment	Diagnosis	NavDx	Adjuvant treatment	Follow up
A	64	M	None	H&N, Anus	No palpable lymphadenopathy. Fullness of right tonsil with mucosal irregularity. Normal appearing base of tongue	CT Neck: bilateral tonsils mildly enlarged, no lymphadenopathy	Bilateral tonsillectomy revealing invasive SCC with positive right tonsil margin Re‐resection of right tonsil bed and bilateral neck dissections	T1N1M0 p16+ SCC	Positive shortly after presentation Negative after initial tonsillectomy	—	q3–4 month surveillance until 2 years post‐surgery, then every 6 months until 5 years post‐surgery
B	64	M	None	H&N	Normal appearing palatine tonsils and base of tongue. No palpable lymphadenopathy.	CT neck: prominent left level 2A lymph node. PET/CT: single hypermetabolic left level 2A lymph node, no identifiable primary in the head and neck Left cervical LN biopsy showing p16+ SCC	Left radical tonsillectomy with left completion selective neck dissection	T1N1M0 p16+ SCC	Negative before radical tonsillectomy	—	Clinical surveillance (3‐month CT scan pending)
C	49	M	None	H&N	Palpable 2 cm left level 2 firm lymph node. Symmetric slightly enlarged tonsils bilaterally without obvious ulceration.	PET/CT: asymmetrically enlarged FDG‐avid left palatine tonsil. Hypermetabolic left level 2A lymph node FNA of left level 2A lymph node: nondiagnostic	Left radical tonsillectomy and left neck dissection	T2N1M0, p16+ SCC	Positive shortly after presentation	Adjuvant radiation	—

### 2.1 Case 1

Patient A was a 64‐year‐old male who underwent Galleri testing in 2023 through his primary care provider, with results indicating a cancer signal of head and neck and anal canal origin. Following the positive result, CT of the neck was obtained and demonstrated bilateral tonsillar hypertrophy. The patient attributed his symptoms to a concurrent acute viral illness and elected observation. He presented to the otolaryngology clinic approximately 6 months later for further evaluation of the positive MCED result. He had a past medical history significant for coronary artery disease and carotid artery stenosis. Review of systems was negative, and social history was notable only for nightly alcohol use. Family history was significant for breast and prostate cancer.

Physical examination at the initial visit demonstrated fullness of the right tonsil with mucosal irregularity, redemonstrated on flexible laryngoscopy. NavDx testing was obtained and returned positive. The patient subsequently underwent bilateral tonsillectomy, with surgical pathology demonstrating invasive HPV‐mediated (p16+) squamous cell carcinoma in both tonsils. The right tonsil tumor extended to the resection margin, and the closest left tumor margin measured 1 mm.

He underwent revision right tonsillectomy with resection of the tonsillar bed and bilateral neck dissection. Final surgical pathology demonstrated no residual carcinoma at the re‐resection bed and one positive lymph node in the right neck, consistent with T1N1M0 p16+ disease. He is currently enrolled in active surveillance with clinical follow‐up every 3–4 months through 2 years postoperatively, extending to every 6 months through 5 years. PET imaging obtained 1 year postoperatively demonstrated no FDG‐avid disease, and NavDx testing at the same timepoint was negative.

### 2.2 Case 2

Patient B was a 64‐year‐old male who underwent Galleri testing through his primary care provider in 2025, with results indicating a cancer signal of HPV‐associated head and neck origin. Past medical history was notable for prior basal cell carcinoma of the nasolabial fold and squamous cell carcinoma of the thigh. Review of systems was negative, and social history was notable only for nightly alcohol use.

Physical examination at the initial visit demonstrated normal‐appearing tonsils and no palpable cervical lymphadenopathy. Flexible laryngoscopy was within normal limits. CT of the neck demonstrated a prominent left level 2A lymph node. PET scan confirmed a single hypermetabolic left level 2A cervical lymph node without an identifiable primary lesion in the pharynx or elsewhere in the head and neck. NavDx testing was negative. Cervical lymph node biopsy of the hypermetabolic node was performed and demonstrated p16+ squamous cell carcinoma, consistent with a diagnosis of squamous cell carcinoma of unknown primary.

He underwent left radical tonsillectomy with left completion selective neck dissection. Final surgical pathology demonstrated squamous cell carcinoma of the left tonsil with negative margins, and all dissected neck nodes were negative. Final staging was pT1N1M0 p16+. He is currently enrolled in active surveillance with clinical follow‐up and cross‐sectional imaging every 3 months.

### 2.3 Case 3

Patient C was a 49‐year‐old male who underwent Galleri testing through his primary care provider in 2026, with results indicating a cancer signal of HPV‐associated head and neck origin. He had no significant past medical or surgical history. Review of systems was otherwise unremarkable.

Physical examination at the initial visit demonstrated a palpable, firm 2 cm left level 2 cervical lymph node with symmetric bilateral tonsillar enlargement and no mucosal ulceration. Flexible laryngoscopy redemonstrated asymmetric left tonsillar fullness. PET scan confirmed an asymmetrically enlarged, FDG‐avid left palatine tonsil with an FDG‐avid left level 2A cervical lymph node suspicious for nodal metastasis. NavDx testing was obtained and returned positive.

Fine needle aspiration of the left cervical lymph node was nondiagnostic. He subsequently underwent left tonsillectomy via transoral robotic surgery and left selective neck dissection. Final surgical pathology demonstrated squamous cell carcinoma of the left tonsil with negative margins and one positive left cervical lymph node, consistent with a final staging of pT2pN1cM0 p16+ oropharyngeal squamous cell carcinoma. He is currently receiving adjuvant radiation therapy as part of an ongoing clinical trial.

## 3 Discussion

This case series describes three patients in whom MCED testing prompted otolaryngologic evaluation that ultimately led to the diagnosis of HNSCC. In each case, patients presented for evaluation of HNSCC solely due to a positive MCED test. None of the three male patients included in this case review reported having symptoms indicative of HNSCC including weight loss, dysphagia, dysphonia, hemoptysis, oral pain/ulceration, or neck masses. Two out of the three patients had no clinical evidence of disease after thorough physical examination and laryngoscopy. Imaging findings varied across patients: bilateral tonsillar hypertrophy was identified on CT neck in one patient, isolated cervical lymphadenopathy in a second, and FDG avidity within tonsillar tissue and a cervical lymph node on PET/CT in the third. After undergoing diagnostic and therapeutic interventions, all patients were diagnosed with early‐stage p16+ oropharyngeal squamous cell carcinoma ranging from T1N0 to T2N1.

Pooling our findings with those of Fearington et al., consistent patterns emerge across the 8 patients who underwent MCED‐directed head and neck workup [8]. All 5 confirmed HNSCC cases across both series were p16‐positive oropharyngeal squamous cell carcinoma arising from the palatine tonsil. All occurred in male patients and presented at early stage. The majority had absent or subtle clinical findings at the time of evaluation. Notably, NavDx testing demonstrated concordance with MCED findings across both series. In our cohort, NavDx returned positive at initial presentation and became undetectable following surgical resection in one patient. Fearington et al. similarly reported NavDx positivity in their confirmed HNSCC cases. This suggests that HPV‐specific ctDNA assays may serve a complementary role to MCED testing both in confirming a suspected HPV‐associated signal and in monitoring treatment response. Of the remaining three patients who were ultimately not diagnosed with HNSCC (all from Fearington et al. study), one was diagnosed with sarcoma of the thigh and two had no malignancy identified. This highlights that a head and neck cancer signal origin on MCED testing does not guarantee a HNSCC diagnosis and underscores the importance of a structured, systematic workup.

To standardize the evaluation of patients presenting with a head and neck cancer signal origin on MCED testing, we propose a diagnostic algorithm based on the accumulated experience of both series that builds upon previously published frameworks (Figure 1) [8]. Following urgent otolaryngology referral within 2–4 weeks of a positive result, patients should undergo comprehensive head and neck examination including flexible fiberoptic laryngoscopy, followed by cross‐sectional imaging with CT neck with contrast or PET/CT (the latter preferred when no lesion is identified on examination). For signals with HPV‐associated cancer signal origin, complementary NavDx testing should be considered given its established utility in HPV‐driven oropharyngeal cancer. Directed biopsy—whether via cervical lymph node sampling, tonsillectomy, or lingual tonsillectomy—should follow imaging findings. Pathologic analysis should include p16 immunohistochemistry and HPV in situ hybridization. All confirmed diagnoses should proceed to multidisciplinary tumor board review for treatment planning per NCCN guidelines.

**FIGURE 1:**
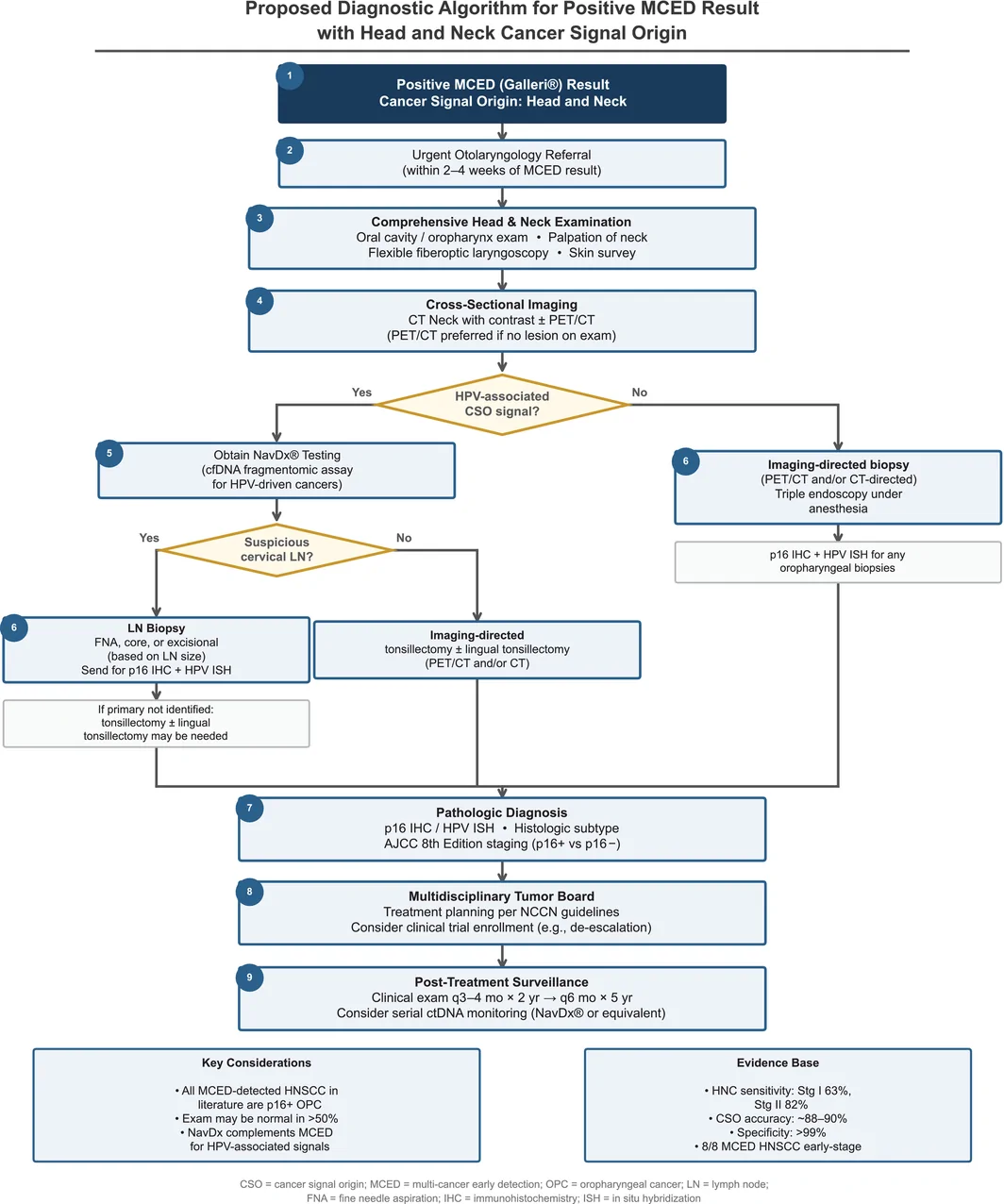
Proposed diagnostic algorithm for otolaryngologic evaluation following a positive multi‐cancer early detection test result with head and neck cancer signal origin.

Four large‐scale clinical studies have evaluated the Galleri MCED test, collectively establishing a strong signal for its utility in head and neck cancer detection. The Circulating Cell‐free Genome Atlas (CCGA) study—a prospective, case‐controlled validation study of over 15 000 participants—demonstrated that head and neck cancer had among the highest sensitivity of any tumor type evaluated, with Stage I sensitivity of 63.2% compared to an all‐cancer average of 16.8%, and Stage IV sensitivity of 96.0%, with cancer signal of origin accuracy of 88.7% across all cancer types [9]. The subsequent PATHFINDER study, the first large‐scale assessment in a clinically relevant screening population of adults aged 50 and older, yielded an overall sensitivity of approximately 29% and a positive predictive value of 38%, though only two oropharyngeal squamous cell carcinomas were identified among true positives, limiting HNSCC‐specific conclusions [4]. PATHFINDER 2 confirmed more robust performance in a real‐world screening population of 23 161 participants, with a positive predictive value of 61.6% and sensitivity of 73.7% among a pre‐specified group of 12 high‐mortality cancers that included head and neck cancer—notably, 75.2% of MCED‐detected cancers lacked established screening alternatives, a category that encompasses HNSCC [10]. Finally, the NHS‐Galleri trial, the first randomized controlled trial of MCED testing, enrolling more than 142 000 asymptomatic participants, did not meet its primary endpoint of a statistically significant reduction in combined Stage III–IV diagnoses. Although the full results of this trial are yet to be published, subsequent analyses have demonstrated a greater than 20% reduction in Stage IV diagnoses for pre‐specified deadly cancers in the second and third screening rounds, suggesting meaningful clinical benefit that accrues with sequential screening and that a characterization of the trial as having simply “failed” does not fully capture.

Beyond their screening role, MCED tests and liquid biopsy technologies hold promise for several additional applications in HNSCC care that warrant prospective investigation. ctDNA clearance during neoadjuvant therapy has emerged as a biomarker of treatment response across multiple malignancies, and similar monitoring via liquid biopsies could provide real‐time molecular indicators to guide therapeutic adaptation in HNSCC prior to surgery [11, 12, 13, 14]. Postoperative minimal residual disease detection represents another compelling application, as ctDNA positivity after definitive therapy has been shown to predict recurrence and precede radiographic relapse by months in other solid tumors—a capability particularly valuable in HNSCC where clinicopathologic features imperfectly guide adjuvant treatment decisions [15]. Finally, as illustrated by Patient B in this series, cancer signal origin prediction may prove useful in the workup of cervical lymph node metastasis from an unknown primary, with tissue‐specific methylation patterns capable of focusing evaluation toward or away from a head and neck origin with approximately 90% accuracy [4, 10].

Several limitations of this study should be acknowledged. This case series includes only three patients from a single institution and therefore cannot establish test performance characteristics, generalizability, or clinical utility. All three patients were male, limiting conclusions about MCED performance across sexes. Referral bias is also possible, as patients presenting to otolaryngology after a positive MCED result may represent a subset enriched for true positives, and those with false positive results or negative workups may not have been captured. Finally, given that all three patients were ultimately diagnosed with p16+ oropharyngeal squamous cell carcinoma, these cases do not address the utility of MCED testing in HPV‐negative disease. Larger prospective studies will be necessary to determine the clinical role of MCED testing specifically for HNSCC detection and management.

## 4 Conclusion

MCED testing represents a promising but still evolving approach to cancer detection. The cases presented here demonstrate that a positive MCED result can prompt evaluation leading to early‐stage HNSCC diagnosis in asymptomatic patients—a clinical scenario that existing screening paradigms are currently unable to address. While current evidence does not support broad population‐level MCED screening, the comparatively high assay sensitivity for head and neck cancer, the absence of alternative screening tools, and the well‐established survival benefit of early‐stage diagnosis collectively suggest that HNSCC may represent a particularly compelling context for targeted MCED application. Prospective studies evaluating MCED testing in high‐risk HNSCC populations are warranted, and otolaryngologists should be prepared to evaluate and manage patients referred on the basis of a positive MCED result.

## Data Availability

The data that support the findings of this study are available on request from the corresponding author. The data are not publicly available due to privacy or ethical restrictions.

## References

[1] L. Q. M. Chow , “Head and Neck Cancer,” New England Journal of Medicine 382, no. 1 (2020): 60–72, 10.1056/NEJMra1715715. 31893516

[2] E. Maghami , N. Ismaila , A. Alvarez , et al., “Diagnosis and Management of Squamous Cell Carcinoma of Unknown Primary in the Head and Neck: ASCO Guideline,” Journal of Clinical Oncology 38, no. 22 (2020): 2570–2596, 10.1200/JCO.20.00275. 32324430

[3] A. Gunning , S. Kumar , C. K. Williams , et al., “Analytical Validation of NavDx, a cfDNA‐Based Fragmentomic Profiling Assay for HPV‐Driven Cancers,” Diagnostics (Basel) 13, no. 4 (2023): 725, 10.3390/diagnostics13040725. PMC9955790 36832208

[4] D. Schrag , T. M. Beer , C. H. McDonnell, 3rd , et al., “Blood‐Based Tests for Multicancer Early Detection (PATHFINDER): A Prospective Cohort Study,” Lancet 402, no. 10409 (2023): 1251–1260, 10.1016/S0140-6736(23)01700-2. PMC11027492 37805216

[5] E. K. O'Donnell , T. L. Kauffman , S. Asnis , et al., “Diagnostic Outcomes Among Patients With Positive Multi‐Cancer Early Detection Test Results,” Cancer Research Communications 6, no. 3 (2026): 511–515, 10.1158/2767-9764.CRC-25-0723. PMC13012066 41721625

[6] M. Matrana , V. Shukla , D. Kingsbury , et al., “Real‐World Data and Clinical Experience From Over 100,000 Multi‐Cancer Early Detection Tests,” Nature Communications 16, no. 1 (2025): 9625, 10.1038/s41467-025-64094-7. PMC12578847 41173830

[7] E. Kennedy , G. Durm , and J. L. Farlow , “Multicancer Early Detection Tests: A State‐Of‐The‐Art Review for Otolaryngologists,” OTO Open 8, no. 4 (2024): e70040, 10.1002/oto2.70040. PMC11512445 39463807

[8] F. W. Fearington , C. Y. Zhao , S. Romero‐Brufau , et al., “Addressing Positive Multi‐Cancer Early Detection Tests in Head and Neck Surgery: Experience With Head and Neck Work Up for High‐Risk Referrals,” Oral Oncology 152 (2024): 106809, 10.1016/j.oraloncology.2024.106809. 38621326

[9] E. A. Klein , D. Richards , A. Cohn , et al., “Clinical Validation of a Targeted Methylation‐Based Multi‐Cancer Early Detection Test Using an Independent Validation Set,” Annals of Oncology 32, no. 9 (2021): 1167–1177, 10.1016/j.annonc.2021.05.806. 34176681

[10] N. Nabavizadeh , C. McDonnell, III , D. Kurbegov , et al., “LBA64 Safety and Performance of a Multi‐Cancer Early Detection (MCED) Test in an Intended‐Use Population: Initial Results From the Registrational PATHFINDER II Study,” Annals of Oncology 36 (2025): S1605.

[11] P. M. Forde , J. Spicer , S. Lu , et al., “Neoadjuvant Nivolumab Plus Chemotherapy in Resectable Lung Cancer,” New England Journal of Medicine 386, no. 21 (2022): 1973–1985, 10.1056/NEJMoa2202170. PMC9844511 35403841

[12] J. V. Heymach , D. Harpole , T. Mitsudomi , et al., “Perioperative Durvalumab for Resectable Non‐Small‐Cell Lung Cancer,” New England Journal of Medicine 389, no. 18 (2023): 1672–1684, 10.1056/NEJMoa2304875. 37870974

[13] M. Provencio , E. Nadal , A. Insa , et al., “Neoadjuvant Chemotherapy and Nivolumab in Resectable Non‐Small‐Cell Lung Cancer (NADIM): An Open‐Label, Multicentre, Single‐Arm, Phase 2 Trial,” Lancet Oncology 21, no. 11 (2020): 1413–1422, 10.1016/S1470-2045(20)30453-8. 32979984

[14] Consortium IST , D. Yee , A. M. DeMichele , et al., “Association of Event‐Free and Distant Recurrence‐Free Survival With Individual‐Level Pathologic Complete Response in Neoadjuvant Treatment of Stages 2 and 3 Breast Cancer: Three‐Year Follow‐Up Analysis for the I‐SPY2 Adaptively Randomized Clinical Trial,” JAMA Oncology 6, no. 9 (2020): 1355–1362, 10.1001/jamaoncol.2020.2535. PMC7378873 32701140

[15] J. Tie , J. D. Cohen , K. Lahouel , et al., “Circulating Tumor DNA Analysis Guiding Adjuvant Therapy in Stage II Colon Cancer,” New England Journal of Medicine 386, no. 24 (2022): 2261–2272, 10.1056/NEJMoa2200075. PMC9701133 35657320

